# Art as a Learning Tool: Medical Student Perspectives on Implementing Visual Art into Histology Education

**DOI:** 10.7759/cureus.5207

**Published:** 2019-07-23

**Authors:** Vincent Cracolici, Ryan Judd, Daniel Golden, Nicole A Cipriani

**Affiliations:** 1 Pathology, University of Chicago Medical Center, Chicago, USA; 2 Medical Education, The University of Chicago Pritzker School of Medicine, Chicago, USA; 3 Radiation and Cellular Oncology, University of Chicago Medical Center, Chicago, USA; 4 Pathology, University of Chicago Medical Centre, Chicago, USA

**Keywords:** histology, art, medical students, education

## Abstract

Creating visual art to teach and learn histologic concepts is uncommon. A pilot visual art program was developed for use in first-year medical student courses that include histology with the hypothesis that creating visual art would subjectively improve the learning process and lead to learner-based personal incorporation of art into in future learning. Prior to the term, volunteers (n=25) were recruited from 89 first-year medical students. The volunteer group was given art supplies and encouraged to draw histologic images in a free-form setting without restrictions. The control group (n=64) consisted of non-volunteers. Pre- and post-term surveys were distributed to all students, of which 72% and 45% completed the surveys, respectively. Regardless of participation, a majority of students viewed art as a valuable tool to learn medicine prior to and following the term (73% and 82.5%, respectively), however less than half admitted to using art to learn medical concepts (42% and 40%, respectively). In the post-term survey, a higher percentage of students in the experimental group stated they will use art to learn medical concepts in the future (75% vs 40.6%). Most students considered art to be a valuable resource to learn concepts in medicine, including all the students who participated in the art program. Based on the number of students who reported intent to change behavior, the initial hypothesis is supported. Many students favor incorporation of visual art into medical education, we believe that creating visual art may be a worthwhile adjunct tool for histology education.

## Introduction

All fields of medicine rely on interpreting visual cues to recognize patterns, make diagnoses, and integrate complex data to deliver meaningful care. For many medical students, the process of recognizing salient visual cues and translating them into meaningful clinical information may be unfamiliar. This is especially true in histology, in which individual students may have little or no prior experience [1]. Learning and practicing flexibility in the interpretation of visual images helps to develop visual intelligence [2]. Although the importance of developing discerning visual acumen as a fledgling physician is critical, no uniform standards exist for bringing these skills to fruition [3].

Visual art has been variably incorporated into medical education for well over a decade and has had success in enhancing empathy, tolerance for ambiguity, and holistic views of patient care [4-13]. Medical students at all levels have taken part in arts-based interventions in order to promote observational skills. Often, exposure to art is passive. For example, students may be taken to museums and taught methods of assessing key visual characteristics in pieces of visual art. Creating visual art to directly teach and learn basic science concepts has been less frequently reported, but has had described successes, particularly in anatomy [14-21]. While learning the visually dense and often foreign field of histology, medical students may struggle with the material if they have limited previous exposure, lack an open attitude toward the content, or if the educational experience in histology is primarily non-interactive, passive didactics [1]. 

Student perceptions of using visual art to learn histology have not yet been reported. This pilot study hypothesized that creating visual art would subjectively improve the learning process and lead to learner-based personal incorporation of art into their future medical education regardless of subject. Additionally, students who partake in an art program may hold more favorable views of the benefit of art in medical education in general.

## Materials and methods

Within our medical school, first-year medical students complete a 21-week term which includes two courses that each require learning histology (physiology and introduction to pathology). Normal histology is the major component of the histologic content in physiology, and abnormal histology is the major component in pathology. During required didactic lectures, histology is taught in detail. There is no scheduled histology laboratory component in the physiology course; instead, there are online self-study modules. There is a required laboratory component within the pathology course. In both, the self-study and required laboratories, students complete interactive online modules including organ-based histologic content. There is no use of microscopes in either course. Instead, all histologic content is viewed as digital photomicrographs, some of which are whole virtual slides that allow panning and zooming (most closely resembling traditional microscopy), and others are static images. Histologic schematic diagrams are also occasionally displayed. Students complete the online modules individually or in groups. Questions regarding the interpretation of histologic images are included on all examinations. Histologic content is revisited during a second-year course with an emphasis on pathology (this second-year course was not included in the study).

**Figure 1:**
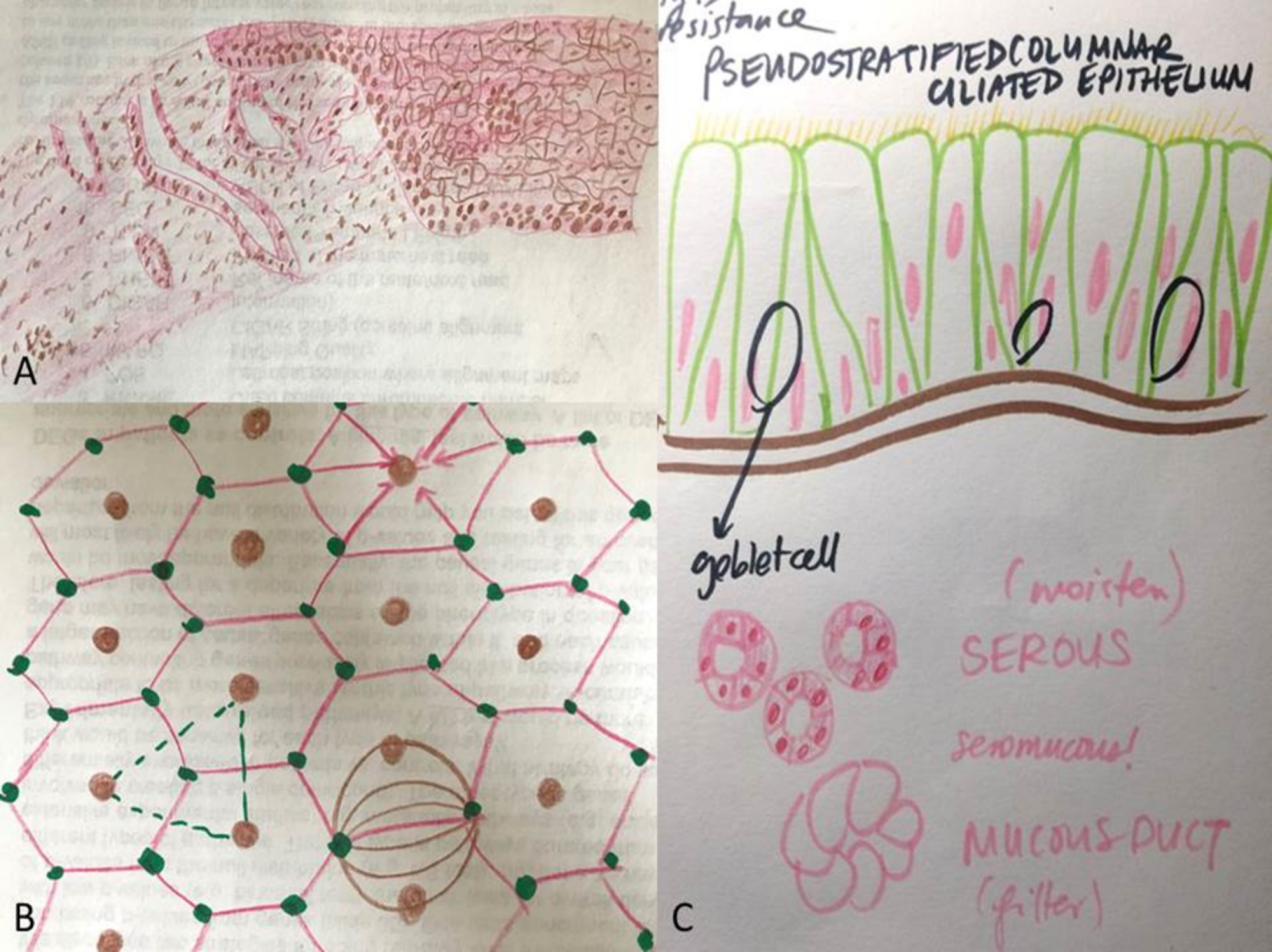
Representative student visual art study pieces depicting histology concepts. These pieces were created in real-time by student participants while concurrently studying the traditional curriculum histology content. Panels A and B courtesy of A. Hung; Panel C courtesy of K. Tran.

Following exemption from the Institutional Review Board (IRB), volunteer students were recruited prior to the initiation of the courses to complete an art intervention in addition to the traditional curriculum (experimental group). The experimental group was given art supplies by the researchers intermittently throughout the term, and encouraged to draw, sketch, or paint histologic images while independently studying the static and dynamic virtual photomicrographs that were required components of the courses (Figure 1). The students could draw the images in whatever way they deemed fit to best enhance their own learning. Participating students were free to use any style or create any quality drawing. Participating students were asked to draw at least a single image for each histology unit encountered over the term, though they could continue to participate throughout the term regardless of the number of drawings produced. Participating students could draw any histologic structure they desired. Students who shared artistic renditions of histology content with the researchers were entered into a raffle to win small prizes. The non-volunteer students completed the traditional curriculum only (control group). Students in the control group were not actively discouraged from drawing histologic images, however the art supplies distributed to the experimental group were not made available to the control group. One volunteer student was designated as the course liaison and recruited peer student participants through direct communication, social media groups, and electronic messaging. The researchers and the course liaison also verbally encouraged continued participation in the art intervention over the duration of the term and assessed the need for specific art supplies, which were regularly obtained from the research team and provided to the students. The art supplies requested by the students were generally basic materials such as colored pencils, markers, crayons, and construction paper. The researchers made efforts to obtain all the requested materials for the participants within practical circumstances. 

Physical pre-term and post-term surveys were distributed in-person to the students during required didactics in order to assess perceptions of art and its use as a learning tool in medicine. The surveys were distributed at the beginning of a didactic session and collected at the end of the same session. The pre-term survey was distributed before announcements of the project or efforts to recruit participants were undertaken. The pre- and post-term surveys were separated by the 21-week term.

The pre-term survey was composed of three statements, each with categorical responses of "Yes", "No", or "Unsure". The pre-term statements were: "I regularly use art to learn concepts in medicine", "I consider myself an artistic person", and "Art is a valuable tool to learn medicine". The post-term survey was identical except for the addition of the fourth statement: "In the future, I will use art to learn concepts in medicine", and also included space for a free-text response for students who participated in the experimental group to describe their experience, provide feedback for the researchers, or leave general comments. The course liaison also encouraged survey responses and solicited participant feedback, as well as assisted the research team in strategically selecting times to distribute materials in order to maximize participation. Statistical analysis was performed using Fisher’s exact and Chi-squared tests (GraphPad Software, La Jolla, CA).

## Results

Overall survey results

A total of 89 first-year medical students completed the 21-week term. Twenty-five (28%) students volunteered to participate in the experimental group, leaving 64 (72%) students in the control group. There were 64 (72% of students) anonymous respondents to the pre-term survey. There were 40 (45% of students) respondents to the post-term survey which included eight (32% of experimental group) students from the experimental group and 32 (50% of control group) from the control group (Table 1).

**Table 1:** Overall Perception of Art as a Learning Tool (Pre- versus Post-Term Survey)

	“I regularly use art to learn concepts in medicine”	“I consider myself an artistic person”*	“Art is a valuable tool to help learn concepts in medicine”	“In the future I will use art to learn concepts in medicine”
Pre-Term Survey (n=64)	Yes: 42%	Yes: 48%	Yes: 73%	Post-Term Survey Only
	No: 48%	No: 41%	No: 8%	
	Unsure: 9%	Unsure: 11%	Unsure: 19%	
Post-Term Survey (n=40)	Yes: 40%	Yes: 27.5%	Yes: 82.5%	Yes: 47.5%
	No: 42%	No: 55%	No: 5%	No: 12.5%
	Unsure: 17.5%	Unsure: 17.5%	Unsure: 12.5%	Unsure: 40%

In the class at large, regardless of participation, most students viewed art as a valuable tool to learn medicine both before and after the term (73% and 82.5%, respectively; p=0.34). Less than half admitted to using art to learn medical concepts prior to and following the course (42% and 40%, respectively; p=0.49). After the course, fewer students considered themselves to be artistic (pre-term 48% and post-term 27.5%; p=0.03). 

 

Post-term survey results: experimental versus control group

In the post-term survey, a similar proportion of students in the experimental and control groups reported using art to learn concepts in medicine (37.5% and 40.6%, respectively; p=0.43). Likewise, the majority of students in the experimental and control groups considered art a valuable tool to learn concepts in medicine (100% and 75%, respectively; p=0.11) (Table 2).

**Table 2:** Post-Term Survey Responses (Experimental versus Control Group)

	“I regularly use art to learn concepts in medicine”	“I consider myself an artistic person”	“Art is a valuable tool to help learn concepts in medicine”	“In the future I will use art to learn concepts in medicine”
Experimental Group (n=8)	Yes: 37.5%	Yes: 50%	Yes: 100%	Yes: 75%
	No: 25%	No: 37.5%	No: 0%	No: 0%
	Unsure: 37.5%	Unsure: 12.5%	Unsure: 0%	Unsure: 25%
Control Group (n=32)	Yes: 40.6%	Yes: 22%	Yes: 75%	Yes: 40.6%
	No: 46.8%	No: 59%	No: 6%	No: 15.6%
	Unsure: 9.3%	Unsure: 18.7%	Unsure: 15.6%	Unsure: 43.7%

At the end of the term, a higher percentage of students in the experimental group stated that they will use art to learn medical concepts in the future (75% vs 40.6%; p=0.10). Students in the experimental group were approximately twice as likely to consider themselves artistic (50% vs 22%, p=0.12). 

Post-term survey results: free-text response

From the experimental group, all (8/8) participating students who responded to the post-term survey answered in the free-text response section. Seven (87.5%) of the students described at least some participation in the project throughout the duration of the term. Five (62.5%) students alluded to time constraints limiting their use of art to study histology and stated that creating images required too much time. Two (25%) students specified spending approximately one hour/week creating images while studying. No other participants specified the amount of time spent creating images. 

## Discussion

This study is among others which have used visual art for basic medical science education, but is in a smaller group focusing on visual art in histology specifically [14,23-25]. It is unique in that it assesses students’ perception of the experience as well as intentions for future behavior. Following completion of the course, most students (82.5%) considered art to be a valuable resource to learn concepts in medicine. Specifically, this includes 100% of the students who participated in the art intervention and 75% of those who did not. Nearly half of the respondents planned to use art in the future to learn medicine (47.5%). The percentage was higher among those who participated in the art program (75% vs 40.6%; p=0.10). The number of students who reported an intent to change behavior suggests that creating visual art may enhance the educational process in histology, and demonstrates that many students favor its incorporation into medical education. This finding is in alignment with other studies which have identified a favorable outlook from students in art interventions and interest in additional future involvement [4,22].

The majority of the class viewed art as a valuable tool to learn histology (82.5%, post-term survey), however, only a portion of the class (40%, post-term survey) regularly used art to learn concepts in medicine. How to ameliorate this discrepancy without creating additional required coursework or curricular changes is a challenge. Incorporating visual art as an elective adjunct avenue for engagement may be an appealing strategy for some medical students. In this case, 28% (25/89) of the class at large volunteered to participate in this art program. In the post-term survey of participants, 100% (8/8) considered art to be a valuable tool to learn medicine, and 75% (6/8) planned to continue to use art to learn concepts in medicine in the future. 

More artistically inclined students may have volunteered to participate in the project. In the post-term survey, 50% of those in the experimental group viewed themselves as artistic compared to 22% of the control group. While some preconceptions may contribute to the differences between the groups, nearly half of the class as a whole, including at least 40% of the control group, plans to use art in the future to learn concepts in medicine. These data suggest that, though art may be preferentially sought out by artistically inclined medical students, many may benefit from the inclusion of art into the curriculum regardless of preconceived notions of artistic skill. Overall, in the pre-term survey, 48% of students consider themselves artistic compared to 27.5% of the students in the post-term survey. It is unclear what may have contributed to the class as a whole viewing themselves as less artistic at the end of the term. Possibilities include a low response rate to the post-term survey (45% of all students), including a low response rate within the experimental group (32%).

Virtual and traditional microscopy are equivalent in studies with respect to histology education, but how the learning process may be perceived by students has not been fully elucidated when using these different modalities [26-27]. The current study begins to describe the impression from the students’ perspective. As histology courses become more digitized, active engagement with content is at risk of becoming more observational and efforts to ensure that digital histology education includes forms of active learning are worthwhile [28]. Some instructors have described successes with actively engaging students using a combination of interactive virtual slides, static photomicrographs, small-group work, and interactive questions [28]. Taken together, these methods ensure that students learn how to “read” a slide, not memorize a specific image [29]. Creating visual art may be another tool which can lead students to understanding and identifying the features that define a particular image as representative of an entity. The power of digital photomicrographs in histology education lies in their realistic nature, though their abundant detail may actually impede learning [30]. For initial instruction and learning how to “read” a slide, there is value in sketching a complex visual field in order to focus on the important elements, eliminate the “noise”, and derive meaning from the image. Creating visual art can assist in digital histology education by serving as an interactive process and be a mechanism to avoid the burden of “gratuitous detail” [30].

One barrier toward continued participation in the art program may be concerns for a lack of time among students, as noted in the free-text response section of the post-term survey. A recent publication evaluated the efficacy of self-body painting to learn anatomy [23]. In this study, students who created art positively reflected on the experience and viewed the opportunity as a respite from traditional didactics [23]. Given the experimental design used by these authors, the student participants in the painting arm of the study were given less time than their peers to “study” for the associated examination yet still performed as well. This may assuage the students in the present study whose primary criticism centered on too great of a time commitment to create pieces and suggests that the time investment of drawing may allow for more rapid or efficient learning. 

Limitations of this study include a relatively low post-term survey response (45% overall), particularly among participants in the experimental group (32%). A greater proportion of respondents may clarify or strengthen the conclusions in this study. Also, the pre-term survey was distributed to the class at-large without knowledge of which respondents ended up in the experimental group versus the control group. Although group-specific data are available for the post-term survey, the ability to assess for selection bias and comment on temporal changes within each group is limited. The lack of randomization into participating and non-participating groups at the initiation of the study allows for the possibility of selection bias and precludes complete certainty that the students who participated were academically equivalent to their peers. Though the lack of randomization may somewhat undermine our ability to assess the value of an art program to objectively improve learning, we feel that the inclusion of visual art into a histology curriculum may act as one adjunct device to facilitate the education of a diverse group of students with varying educational preferences and encourage some otherwise less artistically inclined students to develop new skills. Due to medical school regulations for student protection and anonymity, student performance on exams can not be reported here. Students were unable to be followed to subsequent courses to determine whether those anticipating use of art in future courses actually used art. Finally, this is a single-site, pilot study, therefore the results of which may not be immediately generalized to other institutions. 

After completing this pilot study, many future directions are possible and may include reproducing this experience in other visually oriented areas of medicine such as radiology or advanced pathophysiology. The effect of drawing for individuals with a clear interest in learning histology (such as pathology residents) might also illustrate how the educational process can be augmented with visual art. To more rigorously examine the effects of visual art in histology education and better control for selection bias, a class-wide randomization into participating and non-participating groups would be ideal. Finally, assessing the exam grades of students who draw images compared to those who might not further illuminate the effectiveness of drawing in learning.

## Conclusions

Overall, most students considered art to be a valuable tool to learn medicine (both before and after the course and in the experimental and control groups). Most students who participated in the arts-based intervention (75%) stated that they would use art in the future to learn concepts in medicine, while a lower percent of students in the control group (40%) intends to use art in the future. Student interest in visual art and creating art to aid in learning material may be reinforced by a program such as the one described here. Further long-term studies to evaluate the use of art in histology and medical education are warranted.
